# Clinical value of propranolol combined with oxaliplatin and tigio in concurrent chemoradiotherapy for locally advanced gastric cancer

**DOI:** 10.12669/pjms.38.5.5311

**Published:** 2022

**Authors:** Yong Chen, Yu Chen, Zhibang Wang, He Li, Yongqi Wang

**Affiliations:** 1 Yong Chen, Department of Gastrointestinal Oncology, The First Affiliated Hospital of Hainan Medical University, Haikou 570102, Hainan, China; 2 Yu Chen, Department of General Surgery, The First Affiliated Hospital of Hainan Medical University, Haikou 570102, Hainan, China; 3 Zhibang Wang, Department of Colorectal Surgery, Hainan Cancer Hospital, Haikou 570102, Hainan, China; 4 He Li, Department of General Surgery, The First Affiliated Hospital of Hainan Medical University, Haikou 570102, Hainan, China; 5 Yongqi Wang, Department of Gastrointestinal Oncology, The First Affiliated Hospital of Hainan Medical University, Haikou 570102, Hainan, China

**Keywords:** Gastric Cancer, Locally Advanced, Concurrent Chemoradiotherapy, Propranolol

## Abstract

**Objectives:**

To investigate the clinical value of propranolol combined with oxaliplatin and tigio in concurrent chemoradiotherapy for locally advanced gastric cancer.

**Methods:**

A total of 74 patients with locally advanced gastric cancer admitted to the First Affiliated Hospital of Hainan Medical University from August 2018 to June 2020 were selected as the subject and divided into two groups by random number table method: Group-A and Group-B, with 37 cases in each group. Patients in Group-A were treated with oxaliplatin injection and oral administration of tigio combined with concurrent radiotherapy, while patients in Group-B were given propranolol on the basis of treatment in Group-A. The clinical efficacy and incidence of adverse reactions in the two groups were observed.

**Results:**

The response rate (RR) of Group-B was higher than that of Group-A, but with no statistically significant difference (P>0.05). No statistical difference was observed in the incidence of gastrointestinal reaction, bone marrow suppression, oral mucositis, and the incidence of grade III-IV adverse reactions in the two groups (P>0.05). There were no serious adverse reactions related to propranolol in Group-B, and the levels of tumor markers CEA, CA50, CA125, and CA242 in Group-B were lower than those in Group-A.

**Conclusion:**

Propranolol combined with oxaliplatin and tigio boasts satisfactory sensitization safety in radiotherapy for gastric cancer, but its sensitization effect needs to be further investigated in a multi-center study involving large sample size.

## INTRODUCTION

Gastric cancer, as a common type of malignant tumor, ranks fourth among the most common tumors in the world. More than 900,000 gastric cancer patients were newly diagnosed worldwide in 2012, of which 42% were in China.^1^ Early gastric cancer has a 5-year survival rate of up to 90% after radical resection, but lacks specific symptoms. Most patients are diagnosed with gastric cancer in the middle and advanced stages, so local tumor progression cannot be controlled only by surgery or chemotherapy. Radiotherapy is a satisfactory means of local area control, and various radiotherapy methods such as preoperative radiotherapy, postoperative radiotherapy and palliative radiotherapy can benefit patients with gastric cancer. However, radiation-tolerant cells are prevalent in tumors, which are important factors for recurrence or metastasis of gastric cancer.^2^,^3^ Propranolol, falling into β-adrenergic blockers, specifically blocks β1 and β2 adrenergic receptors. It is the first-line treatment of hypertension, and is mainly used for the treatment of atrioventricular premature beat, sinus tachycardia, atrial fibrillation and so on. It has been shown in recent studies that propranolol has a radiotherapy sensitization effect in basic studies, but there is no clinical report of propranolol used for for radiotherapy sensitization in patients with gastric cancer. For this reason, further studies need to be carried out to investigate the effect of propranolol in clinical application[4]. In this study, propranolol was used in patients with gastric cancer who underwent concurrent chemoradiotherapy to preliminarily observe the clinical effect of propranolol as sensitizer in the treatment of gastric cancer to see its clinical value.

## METHODS

A total of 74 patients with locally advanced gastric cancer admitted to the First Affiliated Hospital of Hainan Medical University from August 2018 to June 2020 were selected as the subject.

### Ethical Approval:

The study was approved by the Institutional Ethics Committee of The First Affiliated Hospital of Hainan Medical University, (Date Sept. 3,2021) and written informed consent was obtained from all participants.

### Inclusion criteria:

- Patients diagnosed with gastric cancer by endoscopy and pathology;
- Patients confirmed by ECT, CT, MRI, ultrasound, PET/CT and other examinations as locally advanced gastric cancer, refused surgery, had contraindications or no indications for surgery, and were unable to undergo surgical treatment;
- Patients who did not undergo surgery or chemoradiotherapy before surgery;
- Patients who were informed about the study and signed an informed consent.

### Exclusion criteria:

- Patients with severe liver and kidney dysfunction and coagulation mechanism disorders;
- Patients with primary tumors at other sites
- Pregnant or lactating women;
- Patients with diffuse liver invasion, brain metastasis, meningeal metastasis;
- Patients with drug allergy in this study;
- Patients with chronic asthmatic bronchitis or bronchial asthma, sinus bradycardia, sick sinus syndrome, atrioventricular block and other contraindications to the use of propranolol;
- Patients with mental abnormalities, severe cognitive dysfunction, audio-visual language function defects;
- Patients proposed to be included or already included in other clinical studies.

A total of 74 patients were included and divided into two groups by random number table method: Group-A and Group-B, with 37 cases in each group. No statistically significant difference was observed in the comparison of general data such as gender, age, body mass index (BMI), TNM staging, and pathological type of the two groups (P>0.05), indicating comparability. Table-I.

**Table I:** Comparison of general conditions between the two groups.

Group	Number of cases	Gender (male/female, number of cases)	Age (years old)	BMI (kg/m2)	TNM stage (II/IIIa/IIIb/IIIc, number of cases)	Pathological types (highly differentiated/moderately differentiated/poorly differentiated/signet ring, number of cases)
Group-A	37	21/16	58.74±8.26	21.38±2.05	4/11/13/9	8/17/9/3
Group-B	37	24/13	58.08±10.27	21.49±2.36	3/13/13/8	6/17/12/2
t/χ2		0.510	0.874	0.530	0.368	0.914
P		0.475	0.386	0.598	0.947	0.822

Patients in Group-A were treated with oxaliplatin injection, oral administration of tigio combined with synchronous radiotherapy. Specifically, intensity-modulated radiotherapy (IMRTR) was used and routine analysis was performed, 5 times/week, at a dose of 45Gy, 1.8Gy/time, normal tissue limit: 60% liver < 30Gy, unilateral kidney 60% < 20Gy, 30% liver < 50Gy, spinal cord < 45Gy; 130 mg·m^-2^ oxaliplatin (Shanghai Yien Chemical Technology Co., Item No.: R002057, Specification: 100mg) was injected intravenously for > 1.5 hours on the first day, with an interval of sven days and a cycle of 21 days; 40mg/m2 Tigio (Tegafur, Japan Taiho Pharmaceutical Co., Ltd., Registration No.: H20100229, Specification: 20mg) was taken orally, once in the morning and once in the evening, and the drug was stopped for 7d after continuous administration for 14 days, which was taken as one cycle for four consecutive cycles. Patients in Group-B were given propranolol daily during radiotherapy on the basis of treatment in Group-A. The initial dose was 10 mg twice/d, and was increased at 10mg twice a day until 60mg twice a day, or the maximum tolerated dose (basal heart rate decline > 25% or < 55 times/min, or systolic blood pressure < 90mmHg) was achieved. Baseline heart rate and blood pressure were recorded daily during the study period and compared with those before medication.

### Observation Indicators:

Objective response rate (RR) of the two groups were observed. The RR rate was evaluated using the RECIST1.1 Response Evaluation Criteria for Solid Tumors, which was divided into complete response (CR), partial response (PR), stable disease (SD) and disease progression (PD), in which the percentage of patients with CR+PR was the response rate (RR). Adverse reactions during treatment of the two groups were observed according to CTCAE3.0 and RTOG common adverse reaction standards. The dosage of propranolol and related adverse reactions in Group-B were observed. The levels of tumor markers CEA, CA50, CA125 and CA242 after treatment were compared between the two groups.

### Statistical Analysis:

All data in this study were processed by SPSS23.0 statistical software, measurement data were expressed as mean±standard deviation, and a paired t test was performed between the two groups. Measurement data were tested by χ2 test and expressed by rate or percentage. P<0.05 indicates a statistically significant difference.

## RESULTS

The RR rate of Group-B was higher than that of Group-A, but with no statistically significant difference (P > 0.05), as shown in Table-II. No statistical difference was observed in the incidence of gastrointestinal reaction, bone marrow suppression, oral mucositis, and the incidence of grade III-IV adverse reactions in the two groups (P>0.05), as shown in Table-III. Thirty five of thirty seven patients reached the maximum dose of 60mg (twice/d). No significant adverse reactions such as dizziness, bronchospasm, dyspnea and fatigue were observed during the administration, and no hypotension and bradycardia were detected. Two patients had a heart rate drop of 50-60 beats/min when they were administered 30mg ((twice/daily) and 40mg ((twice/daily), and no heart rate reduction was observed after the dose reduction. The levels of tumor markers CEA, CA50, CA125 and CA242 in Group-B were lower than those in Group-A, with statistically significant differences (P < 0.05), as shown in Table-IV.

**Table II:** Comparison of clinical efficacy between the two groups [cases (%)].

Group	Number of cases	CR	PR	SD	PD	RR
Group-A	37	5 (13.51)	13 (35.14)	13 (35.14)	6 (16.22)	18 (48.65)
Group-B	37	7 (18.92)	16 (43.24)	11 (29.73)	3 (8.11)	23 (62.16)
χ2						1.367
P						0.242

**Table III:** Comparison of incidence of adverse reactions between the two groups [cases (%)].

Group	Number of cases	Gastrointestinal reaction		Bone marrow suppression		Oral mucositis	

		Incidence	Incidence of Grade III-IV	Incidence	Incidence of Grade III-IV	Incidence	Incidence of Grade III-IV
Group-A	37	12 (32.43)	2 (5.41)	8 (21.62)	0 (0.00)	4 (10.81)	0 (0.00)
Group-B	37	10 (27.03)	0 (0.00)	5 (13.51)	0 (0.00)	2 (5.41)	0 (0.00)
χ2		0.259	2.056	0.839	-	0.725	-
P		0.611	0.152	0.359	-	0.394	-

**Table IV:** Comparison of CEA, CA50, CA125 and CA242 levels between the two groups (χ¯±S ng/ml).

Group	Number of cases	CEA	CA50	CA125	CA242
Group-A	37	13.28±3.41	24.36±4.17	30.19±5.46	13.17±2.33
Group-B	37	10.24±2.77	19.69±4.23	24.74±4.72	9.18±1.79
t		4.209	4.782	4.593	8.261
P		<0.001	<0.001	<0.001	<0.001

## DISCUSSION

Oxaliplatin, as the third generation of platinum anti-cancer drugs in the treatment of advanced cancer, has the pharmacological action of cross-linking platinum atoms with DNA and antagonizing its replication and transcription with DNA as the target site. Tiggio, as an oral anticancer agent derived from fluorouracil, is commonly used in the treatment of advanced gastric cancer. The combination therapy of the two^5^ boasts of reducing neurotoxicity.

β-adrenergic receptor is a crucial G protein-coupled receptor, and has been proved to increase expression in breast cancer, esophageal cancer, colorectal cancer and other malignant tumors, which has a close bearing on tumor proliferation, invasion, metastasis and recurrence.^6^ In recent years, much attention has been paid to the relationship between β-adrenergic receptors and gastric cancer. It was shown in the study of Wang Youling et al.^7^,^8^ that isoproterenol can activate adrenergic receptors and cause epithelial-mesenchymal transition to promote gastric cancer. In vitro cytology experiments have shown that β-adrenergic receptors are related to the sensitivity of gastric cancer to radiotherapy. Inhibition of β-adrenergic receptor activity may increase the sensitivity of gastric cancer cells to radiotherapy, while activation of β-adrenergic receptors can reduce the sensitivity of gastric cancer to radiotherapy.^9^-^10^ It has also been shown in other studies^11^ that β-adrenergic receptor inhibitors can improve the prognosis of patients with gastric cancer. Studies on patients with HER-2 positive undergoing chemotherapy have shown that β2 receptor activation can give rise to chemotherapy resistance in patients.^12^ Moreover, studies on glioma, non-small cell lung cancer, cervical cancer, and ovarian cancer^13^-^16^ have also shown that β-blockers boast of improving the long-term survival rate of patients.

Propranolol falls into a class of non-selective β blockers commonly used for the treatment of hypertension, supraventricular tachycardia, ischemic heart disease, arrhythmias, etc. Recent studies^17^,^18^ have shown that propranolol has anti-tumor activity in neuroblastoma, and its radiotherapy sensitization effect has also been shown in vitro studies. It has been shown in studies^19^-^20^ that NF-κB mediated signaling pathway is associated with radiation resistance characteristics and adverse clinical outcomes in a variety of cancers, and reducing the expression of NF-κB and its downstream genes EGFR, COX-2, and VEGF is contribute to improving radiotherapy sensitivity. In the study of Ke Dongping et al.^21^, gastric cancer cell SGC-7901 and BALA/ C-nunu nude mouse subcutaneous transplanted tumor model were used for intervention with propranolol. The results showed that propranolol administration prior to radiotherapy boasts a variety of efficacy, such as inhibiting the expression of NF-κB, EGFR, COX, VEGF and other factors, improving the radiotherapy effect, and achieving radiotherapy sensitization effect. As shown in the results of this study, there was no statistically significant difference in the RR rate of the two groups, but the RR rate of Group-B showed an upward trend. The possible reasons could be:

- Fewer cases were included in this study;
- In vitro experiment and nude mouse transplanted tumor model failed to fully reflect the survival microenvironment of human tumor, and the radiotherapy sensitization effect of propranolol in clinical application may not be as obvious as that in vitro experiment and experimental animals;
- In both in vitro and experimental animal studies, radiation therapy was the preferred treatment for experimental animals. In this study, concurrent chemoradiotherapy was used for patients out of ethical consideration, and the final RR showed the comprehensive effect of radiotherapy + chemotherapy, which affected the radiotherapy sensitization effect to a certain extent. In view of this, a multi-center study involving more cases need to be conducted to further investigate the radiotherapy sensitization effect of propranolol on patients with gastric cancer.

The common adverse reactions of propranolol include dizziness, fatigue, gastrointestinal discomfort, decreased blood pressure, etc.^22^ Generally, such symptoms are mild and can be relieved or disappear spontaneously after continued medication or reduced dose. No serious adverse events such as death or serious heart problems have been reported.^23^ In this study, no statistical significance can be seen in the incidence of gastrointestinal reaction, bone marrow suppression, and oral mucositis in the two groups, suggesting that propranolol did not increase the complications of concurrent chemoradiotherapy. Only two of the 37 patients had reduced heart rate at 30mg (twice a day) and 40mg (twice a day), and their symptoms were relieved after reduced dose, suggesting that propranolol is safe for patients with gastric cancer undergoing radiotherapy.

### Conclusion:

Propranolol boasts satisfactory sensitization safety in radiotherapy for gastric cancer, but its sensitization effect needs to be further investigated in a multi-center study involving large sample size.

### Authors’ Contributions:

**YC &** **YC:** Designed this study and prepared this manuscript,and are responsible and accountable for the accuracy or integrity of the work.

**ZW:** Collected and analyzed clinical data.

**HL &** **YW:** Significantly revised this manuscript.
